# *MED12* Alterations in Both Human Benign and Malignant Uterine Soft Tissue Tumors

**DOI:** 10.1371/journal.pone.0040015

**Published:** 2012-06-29

**Authors:** Gaëlle Pérot, Sabrina Croce, Agnès Ribeiro, Pauline Lagarde, Valérie Velasco, Agnès Neuville, Jean-Michel Coindre, Eberhard Stoeckle, Anne Floquet, Gaëtan MacGrogan, Frédéric Chibon

**Affiliations:** 1 INSERM U916, Institut Bergonié Cancer Institute, Bordeaux, France; 2 Department of Pathology, Institut Bergonié Cancer Institute, Bordeaux, France; 3 Department of Molecular Pathology, Institut Bergonié Cancer Institute, Bordeaux, France; 4 University Victor Segalen, Bordeaux, France; 5 Department of Surgery, Institut Bergonié Cancer Institute, Bordeaux, France; 6 Department of Medical Oncology, Institut Bergonié Cancer Institute, Bordeaux, France; Ospedale Pediatrico Bambino Gesu', Italy

## Abstract

The relationship between benign uterine leiomyomas and their malignant counterparts, *i.e.* leiomyosarcomas and smooth muscle tumors of uncertain malignant potential (STUMP), is still poorly understood. The idea that a leiomyosarcoma could derive from a leiomyoma is still controversial. Recently *MED12* mutations have been reported in uterine leiomyomas. In this study we asked whether such mutations could also be involved in leiomyosarcomas and STUMP oncogenesis. For this purpose we examined 33 uterine mesenchymal tumors by sequencing the hot-spot mutation region of *MED12*. We determined that *MED12* is altered in 66.6% of typical leiomyomas as previously reported but also in 11% of STUMP and 20% of leiomyosarcomas. The mutated allele is predominantly expressed in leiomyomas and STUMP. Interestingly all classical leiomyomas exhibit MED12 protein expression while 40% of atypical leiomyomas, 50% of STUMP and 80% of leiomyosarcomas (among them the two mutated ones) do not express MED12. All these tumors without protein expression exhibit complex genomic profiles. No mutations and no expression loss were identified in an additional series of 38 non-uterine leiomyosarcomas. *MED12* mutations are not exclusive to leiomyomas but seem to be specific to uterine malignancies. A previous study has suggested that *MED12* mutations in leiomyomas could lead to Wnt/β-catenin pathway activation however our immunohistochemistry results show that there is no association between *MED12* status and β-catenin nuclear/cytoplasmic localization. Collectively, our results show that subgroups of benign and malignant tumors share a common genetics. We propose here that *MED12* alterations could be implicated in the development of smooth muscle tumor and that its expression could be inhibited in malignant tumors.

## Introduction

Smooth muscle tumors (SMT) are the most common mesenchymal tumors of the uterus. They encompass leiomyomas (LM), atypical LM, Smooth muscle Tumor of Uncertain Malignant Potential (STUMP) and leiomyosarcomas (LMS) [1]–[3]. LM are benign tumors that represent 70% of hysterectomy specimens for non-cancer related conditions in non-menopausal women. Atypical LM is a LM variant with atypical, unusual nuclei with spotty distribution [4]. STUMP tumors represent a heterogeneous group of rare tumors that cannot be histologically diagnosed as unequivocally benign or malignant, according to the World Health Organization classification [1]–[3]. Uterine LMS are aggressive tumors with a poor prognosis overall, representing 40% of uterine sarcomas and 1–3% of uterine malignancies. The histological distinction between benign and malignant SMT is based on a tree-feature morphological approach encompassing atypia, necrosis and mitotic count proposed in 1994 by Standford investigators [4]. Only a few publications on STUMP and atypical LM are available and they represent a critical problem for pathologists and clinicians at the diagnostic and therapeutic levels respectively. Some studies have tested histological and immunohistochemical tools (Ki-67, BCL2, p16 and p53) [5]–[6] to improve diagnostic process and to evaluate the prognosis of such lesions but unfortunately without clinical utility. Currently LMS are still devoid of therapeutic targets.

The pathogenesis of SMT is poorly understood. It is generally believed that uterine LMS arise *de novo* rather than from any precursor lesions. Nevertheless, some cases of LMS deriving from a pre-existing LM have been described [7]–[15]. Currently, little data is available concerning genetic events that could be implicated in LM development. A few, not specific, genetic alterations occurring infrequently (in around 20% of LM) have been described (chromosome 7q partial deletions, chromosome 12 trisomy, rearrangements of 12q14–15, 6p21–23 for example) (reviewed in [16]). Recently Makinen *et al*. reported recurrent and frequent *Mediator complex subunit* 12 (*MED12*) mutations in uterine LM [17]. Makinen *et al*.'s study is the first report of such frequent alterations identified in 70% of LM. All mutations are located in the intron 1 and exon 2 of *MED12* (6.2% and 64.4% respectively) and are assumed to be activating mutations. The Mediator complex consisting of 26 subunits, seems to be implicated in transcription regulation and act as a bridge between DNA binding transcription factors and the RNA polymerase II initiation complex as reviewed in [18]–[19]. A subcomplex of the Mediator complex, named CDK8 submodule, has been identified and is composed of CDK8, MED12, MED13 and Cyclin C. Several studies have suggested that this subcomplex can either activate or repress transcriptional expression depending on the cellular context as reviewed in [18]–[19].

In the present study, we thus asked whether *MED12* mutations could also be involved in oncogenesis of LM malignant counterparts, *i.e.* LMS and STUMP. To extend the analyses we also assessed *MED12* expression at mRNA and protein levels and studied tumor genomic profiles and β-catenin localization according to MED12 alterations.

## Results

### Are *MED12* mutations exclusive to human uterine leiomyomas?

**Figure 1:**
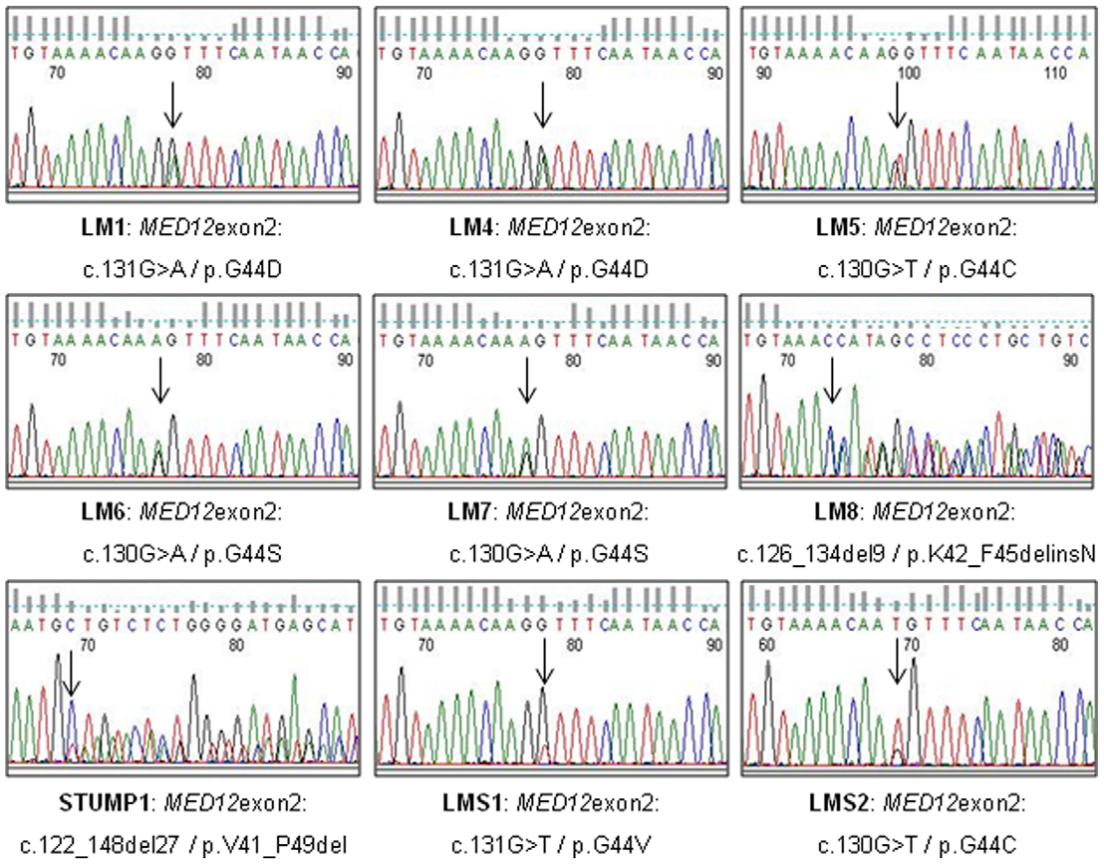
*MED12* genomic mutations Sequence chromatograms showing *MED12* mutations observed on genomic DNA in the nine mutated uterine LM, STUMP and LMS (Sequence viewer: FinchTV, Geospiza). Arrows indicate mutation sites.

To assess this issue we sequenced the mutation hot-spot region of *MED12* described by Makinen *et al* [17] in 33 uterine tumors including nine LM, five atypical LM, nine STUMP and ten LMS. These tumors came from 32 individual patients. All sequences were interpretable and we detected nine mutations (27%) summarized in Table 1 and Figure 1. All tumors displayed only one mutation and all *MED12* mutations are heterozygous as described by Makinen *et al*. [17].

**Table 1:** Summary of *MED12* mutations observed in the series of 33 uterine tumors.

Tumor name	Location	Mutation	Nucleotide change	Predicted protein change
**LM1**	Exon 2	G>A	c.131G>A	p.G44D
**LM4**	Exon 2	G>A	c.131G>A	p.G44D
**LM5**	Exon 2	G>T	c.130G>T	p.G44C
**LM6**	Exon 2	G>A	c.130G>A	p.G44S
**LM7**	Exon 2	G>A	c.130G>A	p.G44S
**LM8**	Exon 2	9 bp del	c.126_134del9	p.K42_F45delinsN
**STUMP1**	Exon 2	27 bp del	c.122_148del27	p.V41_P49del
**LMS1**	Exon 2	G>T	c.131G>T	p.G44V
**LMS2**	Exon 2	G>T	c.130G>T	p.G44C

WT: wild-type, MUT: mutated, bp: base-pair, LM: leiomyoma, STUMP: Smooth muscle Tumor of Uncertain Malignant Potential, LMS: leiomyosarcoma.

Regarding typical leiomyomas, we observed six mutations (6/9: 66.6%), among them five point mutations (83.3%) and one nine-base-pair (bp) deletion (16.7%). All point mutations were located in codon 44 (exon 2) and were missense mutations. Typical LM was the most frequently mutated entity, whereas no mutation was detected in atypical LM. Of note, one STUMP (1/9: 11%) (27bp in-frame deletion) and two uterine LMS (2/10: 20%) (point mutations) were mutated. The two point mutations observed in LMS also concerned the codon 44 and the deletion observed in STUMP encompassed this region (deletion of codons 41 to 49). These results indicate not only that *MED12* is frequently mutated in typical LM (66.6%), but also that mutations are not restricted to benign tumors since one STUMP and two highly aggressive LMS were mutated.

### Are *MED12* mutations exclusive to human uterine smooth muscle tumors?

Given the identification of *MED12* mutations in uterine LMS, we asked whether *MED12* mutations could also be observed in LMS from internal trunk and limbs. Consequently, 38 additional non-uterine LMS were submitted to *MED12* sequencing and no mutations were detected. These results tend to show that even if *MED12* mutations are not restricted to benign tumors, they seem to be specific to uterine smooth muscle tumors.

### Which *MED12* allele is expressed?

Over the last 50 years, it has been extensively demonstrated that in females normal cells X-chromosomal genes present a monoallelic expression due to random inactivation of one of the two X chromosomes [20]. Given that the *MED12* gene is located on the X chromosome (Xq13.1) and that all genomic mutations observed are heterozygous, we sequenced cDNA from all studied cases with good enough RNA quality (69/71) to check which allele is expressed.

**Figure 2:**
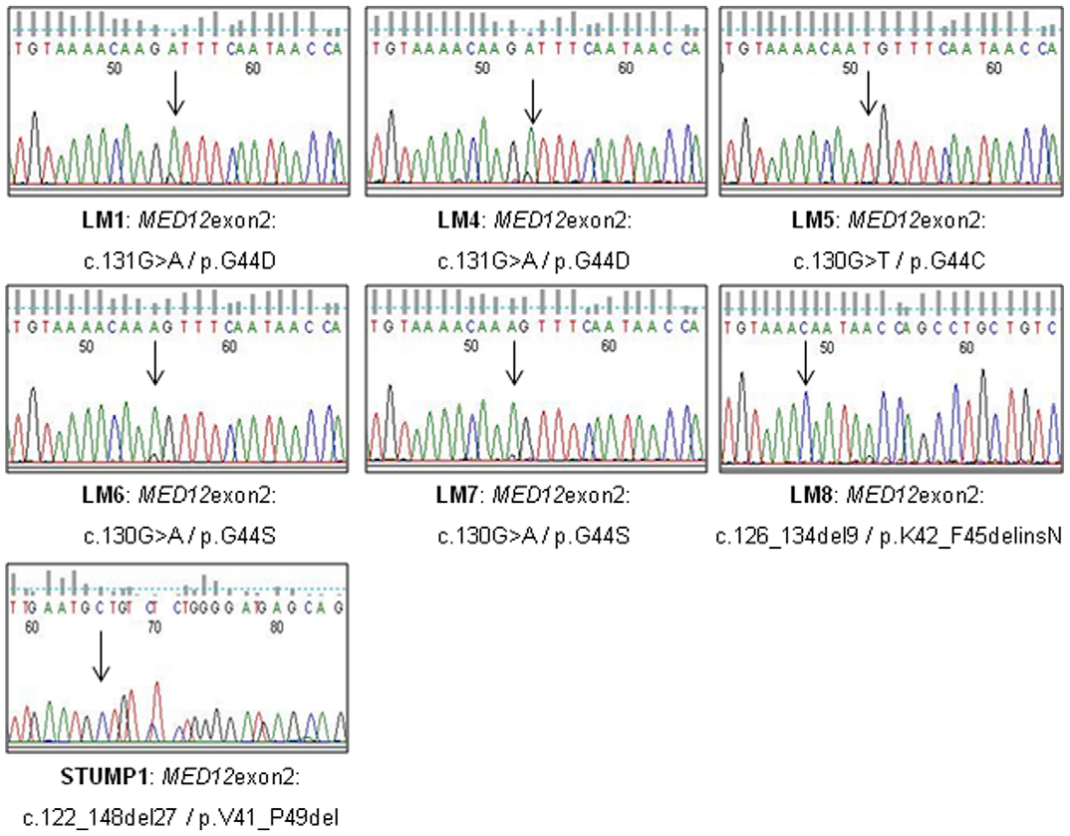
*MED12* mutations on cDNA. Sequence chromatographs of *MED12* mutations observed on cDNA showing that the mutated allele is predominantly expressed. Arrows indicate mutation sites.

In typical leiomyomas, all mutations identified at the genomic level were observed on cDNA (Figure 2). Moreover, in all cases the mutated allele seems to be predominantly expressed (LM1/4/6–8) or seems to be the only one expressed (LM5). We observed the same expression profile for the mutated STUMP (STUMP1). Indeed, only the 27 bp deleted allele seems to be expressed.

**Figure 3:**
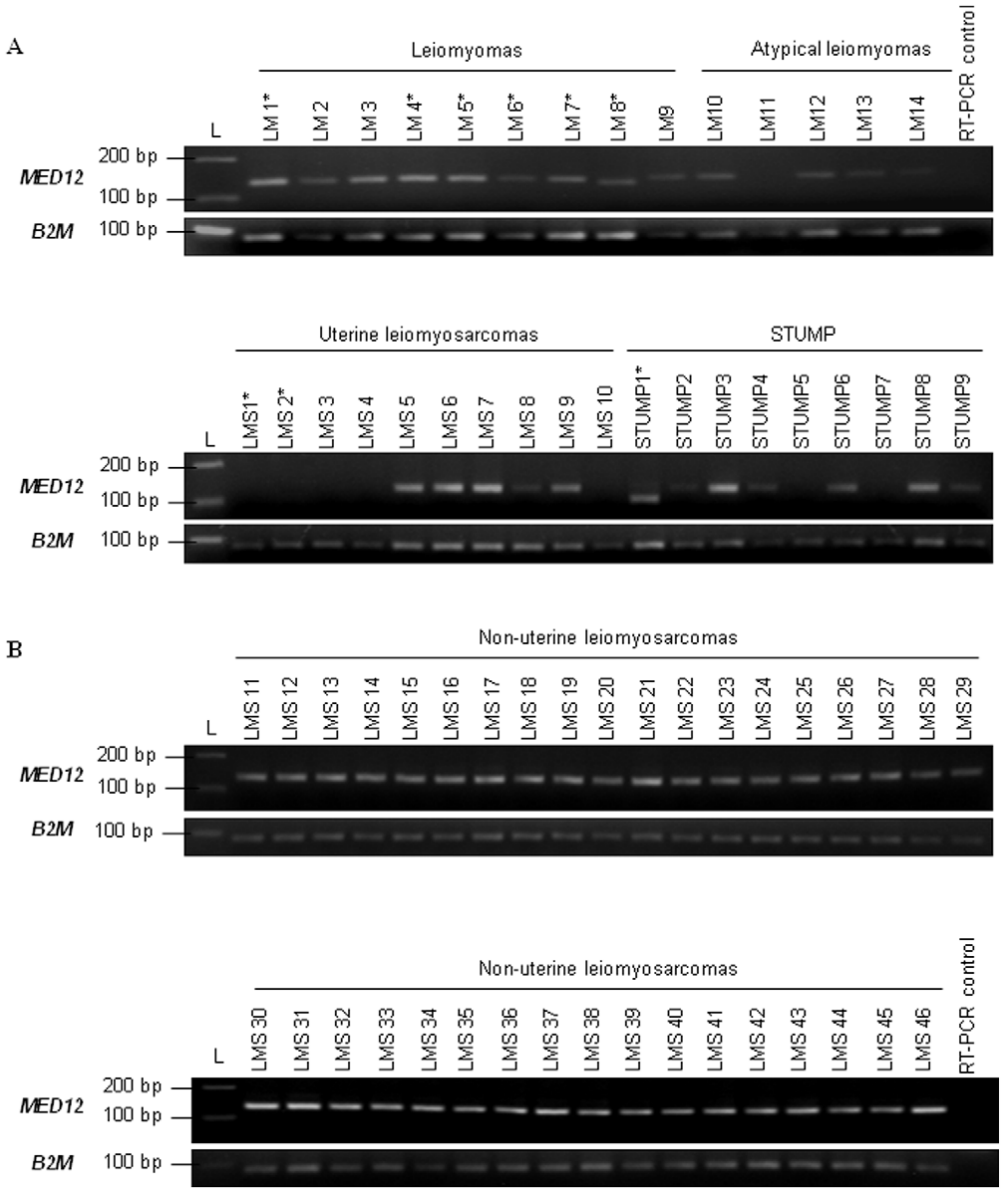
*MED12* RNA expression. (A) Expression profiles of *MED12* and *β2M* (β-2-microglobulin) obtained by RT-PCR in uterine smooth muscle tumors are presented. *β2M* is used as RT-PCR control. (B) Expression profiles of *MED12* and *β2M* (β-2-microglobulin) obtained by RT-PCR in LMS from limbs and internal trunk are presented. *: mutated tumors. L: molecular weight ladder. LM: leiomyoma, LMS: leiomyosarcoma, STUMP: Smooth muscle Tumor of Uncertain Malignant Potential.

Figure 3A presents the RT-PCR products obtained for all uterine tumors studied. We observed that all classical LM express *MED12* (Figure 3A), whereas one atypical LM (LM11, 1/5: 20%) does not exhibit *MED12* expression. β-2-microglobulin control shows a RT-PCR product for all cases (Figure 3A). In the same manner, two STUMP do not express *MED12* (STUMP5 and STUMP7, 2/9: 22.2%). Concerning uterine LMS, no RT-PCR products for *MED12* could be observed in five cases (LMS1 to LMS4 and LMS10, 5/10: 50%). Further, among the five LMS without *MED12* expression there are the two mutated uterine LMS (LMS1 and LMS2).

In order to assess if this *MED12* expression loss could also be observed in LMS from internal trunk and limbs, we performed *MED12* RT-PCR on 36 non-uterine LMS for which frozen material was available (Figure 3B). We observed that all 36 studied LMS display *MED12* expression. Inhibition of *MED12* expression seems to be specific to a subgroup of uterine malignant tumors (STUMP and LMS).

### Is *MED12* protein expressed in uterine tumors?

**Figure 4:**
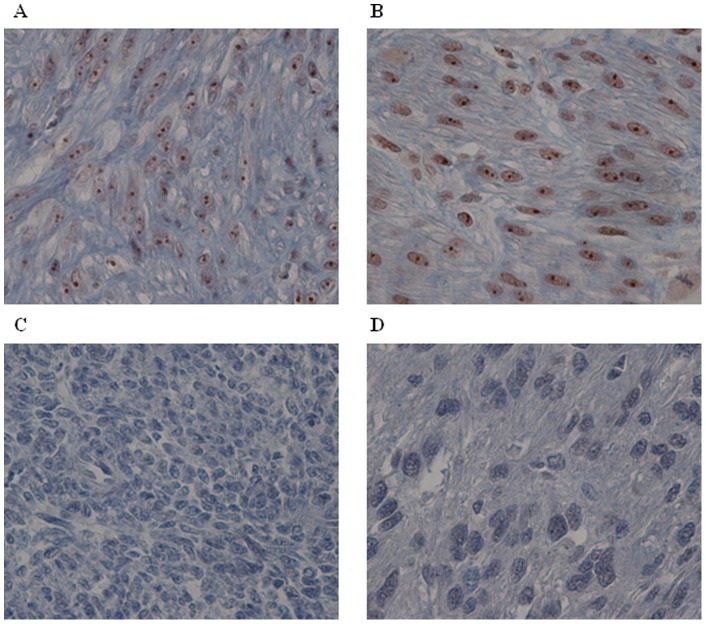
MED12 protein expression. (A) Positive MED12 nuclear labeling in mutated LM6. (B) Positive MED12 nuclear staining in wild-type STUMP8. (C) Wild-type STUMP4 with negative staining (D) Mutated LMS1 without MED12 labeling. Magnification: X40.

In order to confirm *MED12* mRNA expression results at the protein level we performed an immunohistochemistry study. Our results show that all classical LM expressed MED12 protein (Figure 4 and Table 2). In these tumors MED12 is expressed regardless of its mutational status. In contrast, we observed that 40% of atypical LM (2/5), 50% of STUMP (4/8) and 80% of LMS (8/10) do not exhibit MED12 protein expression. All cases with no MED12 mRNA do not present protein expression; those with a slight positivity at the mRNA level exhibit the same negative protein profile and all atypical LM and STUMP with mRNA positivity expressed the protein. Collectively these results not only confirmed mRNA data but also showed that two LMS (LMS5 and LMS6) with mRNA expression do not express MED12 protein.

**Table 2:** *MED12* gene expression and β-catenin localization.

Tumor Name	Histotype	*MED12* status	*MED12* RNA expression	MED12 IHC staining	β-catenin IHC localization
**LM1**	LM	MUT	Positive	Positive	**Negative**
**LM2**	LM	WT	Positive	Positive	M + C
**LM3**	LM	WT	Positive	Positive	C
**LM4**	LM	MUT	Positive	Positive	**Negative**
**LM5**	LM	MUT	Positive	Positive	M
**LM6**	LM	MUT	Positive	Positive	M
**LM7**	LM	MUT	Positive	Positive	M
**LM8**	LM	MUT	Positive	Positive	M
**LM9**	LM	WT	Positive	Focal positivity	M
**LM10**	Atypical LM	WT	Positive	Positive	M
**LM11**	Atypical LM	WT	**Negative**	**Negative**	**Negative**
**LM12**	Atypical LM	WT	Positive	Focal positivity	M
**LM13**	Atypical LM	WT	Positive	Positive	M
**LM14**	Atypical LM	WT	Slight positivity	**Negative**	M + C
**STUMP1**	STUMP	MUT	Positive	Positive	M
**STUMP2**	STUMP	WT	Slight positivity	NA	NA
**STUMP3**	STUMP	WT	Positive	Positive	M + C
**STUMP4**	STUMP	WT	Slight positivity	**Negative**	C
**STUMP5**	STUMP	WT	**Negative**	**Negative**	M
**STUMP6**	STUMP	WT	Positive	Positive	M
**STUMP7**	STUMP	WT	**Negative**	**Negative**	M + C
**STUMP8**	STUMP	WT	Positive	Positive	M
**STUMP9**	STUMP	WT	Slight positivity	**Negative**	M
**LMS1**	LMS	MUT	**Negative**	**Negative**	M + C
**LMS2**	LMS	MUT	**Negative**	**Negative**	M
**LMS3**	LMS	WT	**Negative**	**Negative**	**Negative**
**LMS4**	LMS	WT	**Negative**	**Negative**	M
**LMS5**	LMS	WT	Positive	**Negative**	M
**LMS6**	LMS	WT	Positive	**Negative**	M
**LMS7**	LMS	WT	Positive	Focal positivity	M
**LMS8**	LMS	WT	Slight positivity	**Negative**	C
**LMS9**	LMS	WT	Positive	Positive	M + C
**LMS10**	LMS	WT	**Negative**	**Negative**	C

Tumor histotype and *MED12* mutational status are indicated. LM: leiomyoma, STUMP: Smooth muscle Tumor of Uncertain Malignant Potential, LMS: leiomyosarcoma. WT: wild-type, MUT: mutated. *MED12* mRNA (RT-PCR) and protein expression (IHC) are summarized. Finally β-catenin localization visualized by IHC in tumors is indicated. M: membranous staining, C: cytoplasmic labeling. NA: not available.

### Are *MED12* alterations associated with peculiar genomic profiles?

A recent study has shown that 82.6% of mutated LM do not exhibit genomic alterations and that the remaining 17.4% present very few rearrangements [21]. In contrast another recent study has shown that MED12 alterations are equally distributed among karyotypically normal LM (69%) and uterine leiomyomas with some rearrangements (63%) [22]. In order to ask in our series whether *MED12* alterations could be associated with peculiar genomic profiles we performed array-CGH analysis.

**Figure 5:**
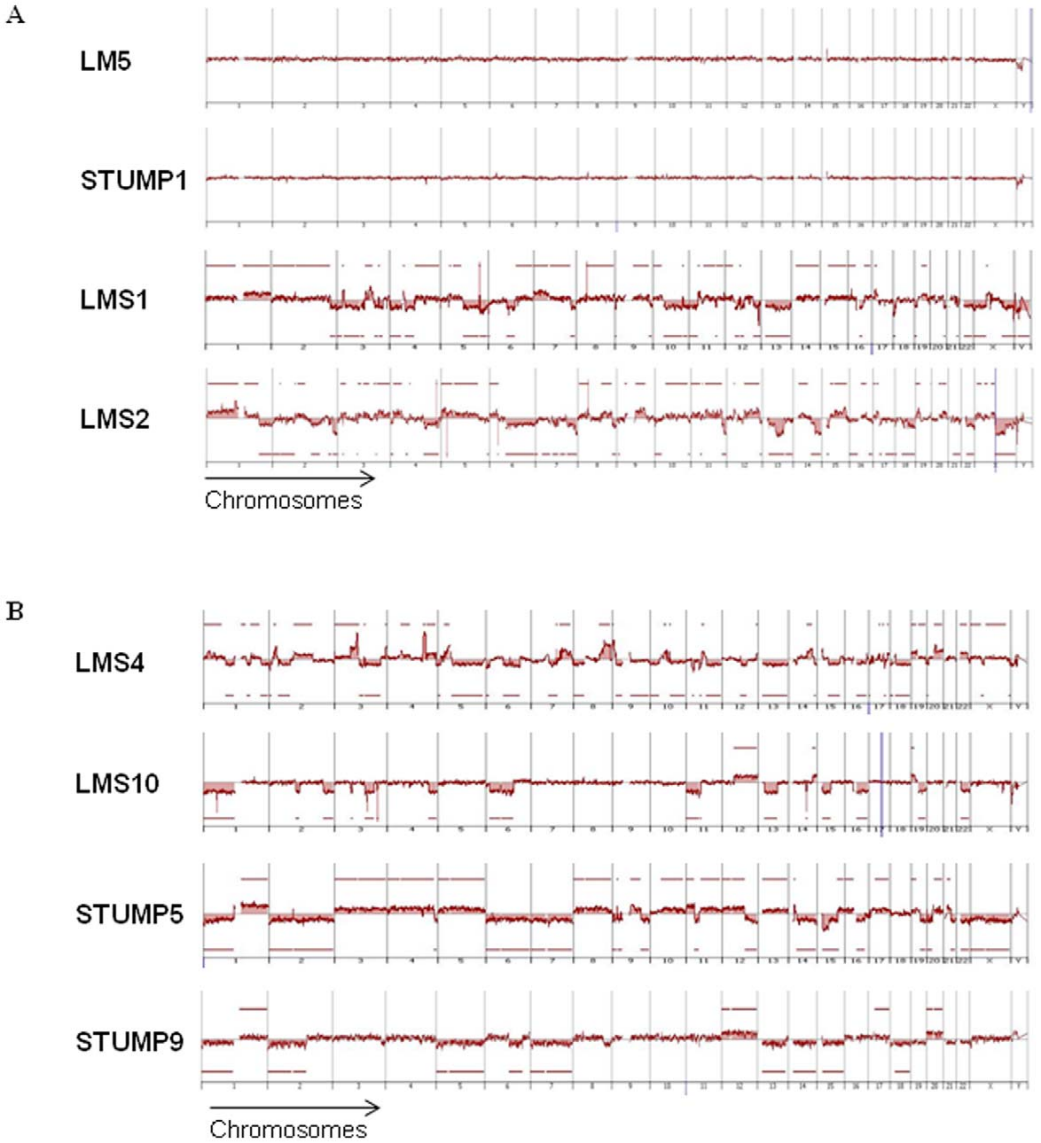
Tumor genomic profiles. (A) CGH profiles of four cases representing a leiomyoma (LM), a STUMP and the two mutated uterine leiomyosarcomas (LMS). (B) CGH profiles of four representative cases without MED12 expression. Genomic alterations are presented and organized from chromosome 1 to 22 and X, Y on the X axis and log2 ratio values are reported on the Y axis. Significant gains or losses are indicated by red lines and red areas above or below each profile, respectively.

Genomic profiles of representative mutated tumors are presented as an example in Figure 5A. All mutated LM present no alterations as is the case for all classical non-mutated LM. The mutated STUMP exhibits a similar profile, whereas the two mutated LMS show lots of chromosome gains and losses. We could thus see that the mutated tumors exhibit the features previously described for their respective histotype [23]–[24]. When we looked at the tumor genomic profiles according to MED12 expression data, we observed that all tumors with no MED12 protein expression exhibit very rearranged genomic profiles. (For example: Figure 5A (LMS1 and LMS2) and Figure 5B). In contrast, wild-type tumors with MED12 expression exhibit no or very few alterations except for LMS7 and LMS9 which are rearranged tumors, as for other LMS.

### Could *MED12* play a role in LM oncogenesis through the β-catenin/Wnt pathway?

**Figure 6:**
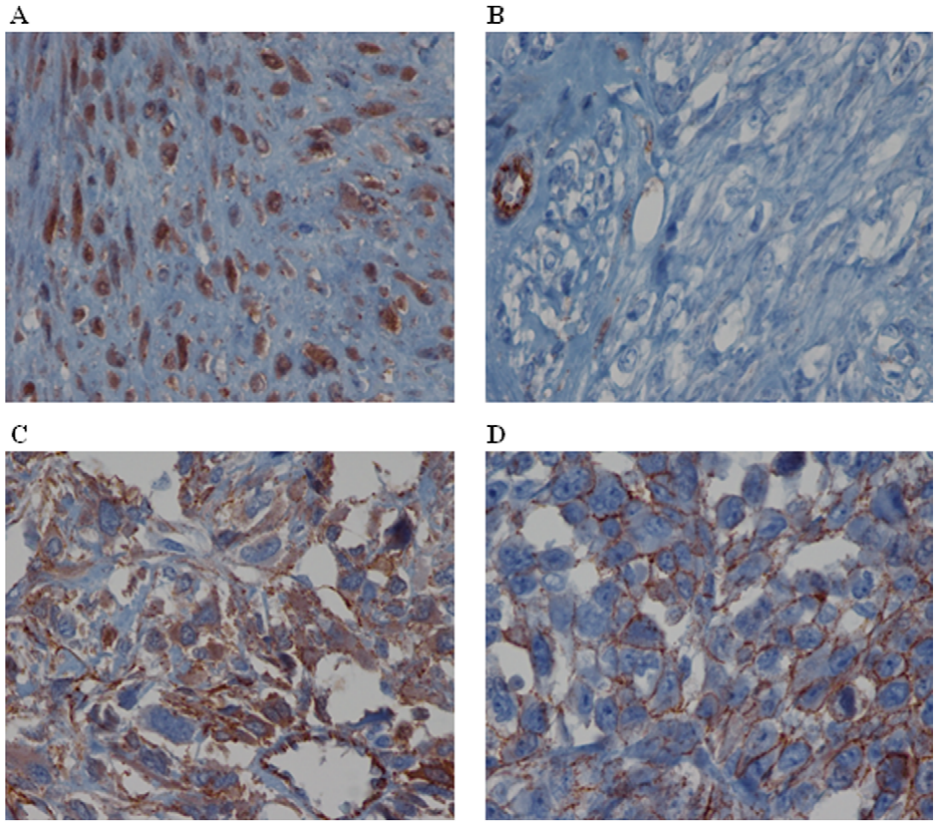
β-catenin expression. (A) Nuclear β-catenin labeling control in a desmoid tumor. (B) Leiomyoma without β-catenin expression (LM1). (C) Uterine leiomyosarcoma with cytoplasmic β-catenin expression (LMS8). (D) Uterine leiomyosarcoma with membranous β-catenin expression (LMS2). Magnification: X40.

Makinen *et al*.'s study has suggested a role of *MED12* mutations in LM through Wnt/β-catenin pathway activation [17] and it has been shown that MED12 is implicated in transcription activation of Wnt target genes by interacting with β-catenin [25]–[26]. We thus assessed the β-catenin expression profile by immunohistochemistry in this uterine tumor series in order to see if the Wnt/β-catenin pathway was activated in these tumors, and if *MED12* alterations were associated with a peculiar β-catenin pattern. We first observed that none of the tumors exhibited nuclear β-catenin, 56.25% of tumors show a membranous staining (18/32), 18.75% present both membranous and cytoplasmic labelings (6/32), 12.5% show only cytoplasmic β-catenin (4/32), and 12.5% are negative for β-catenin (4/32) (Figure 6 and Table 2).

Considering the β-catenin staining in each histotype separately, we saw that 66.6% of classical LM (6/9), 80% of atypical LM (4/5), 87.5% of STUMP (7/8) and 70% of LMS (7/10) display a β-catenin membranous localization. In contrast, a positive cytoplasmic β-catenin labeling, associated or not with a membranous staining, is only observed in 22.2% of classical LM (2/9), 20% of atypical LM (1/5), 37.5% of STUMP (3/8) and 40% of LMS (4/10).

To go further in our analysis, looking at the β-catenin localization pattern according to the *MED12* status, we could observe that 71.4% (5/7) of mutated tumors with MED12 expression display only membranous β-catenin and the remaining 28.6% (2/7) do not show β-catenin expression. The two mutated LMS without MED12 expression both display a membranous staining associated for one of them with a cytoplasmic labeling. Finally concerning the wild-type tumors without MED12 expression, all staining profiles could be observed: 16.7% negative (2/12), 16.7% membranous and cytoplasmic, 25% cytoplasmic (3/12) and 41.6% membranous (5/12). Together, this data suggests that there is no association between *MED12* mutations and cytoplasmic or nuclear β-catenin localization.

## Discussion

Currently the relationship between benign uterine tumors (leiomyomas) and their malignant counterparts, *i.e.* leiomyosarcomas and STUMP, is still poorly understood. The idea that a LMS could derive from a LM is still controversial. Indeed, the large discrepancy in their frequency of occurrence (leiomyosarcomas represent only 0.1 to 0.3% of leiomyomas [9]) could lead to thinking that malignant transformations of LM if they exist are very rare. Up until now, most cases of uterine LMS have been believed to arise *de novo*, although several cases of uterine LMS arising in pre-existing LM have been reported [7]–[12]. In the same manner, some studies have hypothesized that, in the case of LMS with a benign leiomyomatous area, the benign component could be a precursor lesion to LMS [13]–[15]. All these issues could be conciliated by the hypothesis which suggests that only a subset of LM, with variant histological features and/or genomic alterations, have potential for malignant progression, and that these peculiar LM may represent a premalignant transitional state, while most LM have no malignant potential as reviewed in [16].

Recently, recurrent mutations of the *Mediator Complex Subunit 12* gene (*MED12*) have been identified in 70%, 58.8%, 67,6% and 52.2% of LM [17], [21]–[22], [27]. In the present study we have assessed *MED12* gene status in 33 uterine tumors including nine LM, five atypical LM, nine STUMP and ten LMS, and we show not only that 66.6% of LM present *MED12* mutations, confirming previous results, but also that 11% of STUMP and 20% of uterine LMS present *MED12* mutations. In this series all mutations concerned the *MED12* intron1-exon 2 hot-spot region previously described [17], [21]–[22], [27]. Our results show more codon 44 mutations (83.3%) than observed by Makinen *et al*. (49%) and Je *et al*. (66.6%), fewer than observed by Markowski *et al*. (95.8%) but are closed to those of McGuire *et al*. (89.5%). These discrepancies could be due to the various sizes of the series (9, 67, 225, 80 and 148 LM respectively), but more probably to the tumor sampling. Indeed in the present series the nine LM came from nine individual patients as for McGuire *et al*. series which contained only individual samples while in Makinen *et al*.'s series the 225 LM derived from 80 different patients, and in Markowski *et al*.'s series the 80 LM came from 50 distinct patients. We did not observe any codon 36 or 43 point mutations and no intron 1 mutations in our series, which are three other mutated regions identified by Makinen *et al*. The two deletions in our series (9 bp in LM8 and 27 bp in STUMP1) are in-frame as all reported insertions-deletions [17], [21]–[22], [27]. The 27 bp deletion was previously described by McGuire *et al.* [22], and the 9 bp deletion has not been already published [17], [21]–[22], [27]. Both deletions encompass the codons 43 and 44.

In our study we observed two uterine leiomyosarcomas exhibiting a *MED12* mutation. Recently Je *et al*. have published a mutational study of *MED12* in 1862 samples, including leiomyomas, diverse carcinomas, leukemias, sarcomas and other stromal tumors, in which they observed only one malignant tumor with a *MED12* mutation [27]. Among the studied tumors, there were five uterine LMS and the authors observed no *MED12* mutation in these samples as in other sarcomas. These discrepancies may be due to the sampling size indeed in our study we observed a *MED12* mutation only in 20% of uterine LMS. According to Je *et al*. results combined to ours it appears that *MED12* mutations are not exclusive to benign malignancies and are not specific to mesenchymal tumors even if they seem rare in malignant tumors.

The first point thus assessed here is the potential filiation between benign and malignant uterine tumors. Our results show that mutations are not restricted to benign tumors since two highly aggressive LMS (20%) and one STUMP (10%) are mutated.

At least two mechanisms could explain the occurrence of *MED12* mutations in the three entities (LM, STUMP and LMS): either common genetics at the initial developmental steps, or mutated STUMP/LMS were first LM then subsequently acquired alterations leading to malignant evolution. The second hypothesis is consistent with the previously mentioned hypothesis which proposed that a subgroup of LM could undergo malignant transformation and could thus evolve in LMS [7]–[15]. However no such conclusions could be made for the non-mutated uterine LMS on the basis of this data.

We also investigated *MED12* allele expression by RT-PCR, sequencing and immunohistochemistry. All typical leiomyomas expressed *MED12* at mRNA and protein levels and in mutated LM it seems that the mutated allele is predominantly expressed, as described previously [17], [21]–[22]. For cases with a minor wild-type transcript expression, we could hypothesize that it may be due to normal cell contamination. However, we could not exclude a *MED12* wild-type allele expression for a minority subclone of the tumor. Indeed, many studies have described a clonal origin of LM [28]–[31] but one study has shown that some LM could be heterogeneous [32]. Concerning tumors with intermediate or high malignancy we observed that 40% of atypical LM, 50% of STUMP and 80% of LMS do not express MED12 protein. Among these 14 tumors with no MED12 protein 57.1% (8/14) do not exhibit mRNA, 28.6% (4/14) show a weak RT-PCR positivity and 14.3% (2/14) express mRNA. According to array-CGH results all tumors exhibit two copies of the gene. So it seems that *MED12* expression loss is not due to a deletion of the *MED12* allele on the active X chromosome. In tumors with no mRNA and no protein, *MED12* should thus be transcriptionally inhibited. To our knowledge, no data concerning *MED12* expression regulation is currently available so we could only hypothesize that its expression could be inhibited by promoter or histones methylation, or that a transcriptional repressor of *MED12* could be expressed in these tumors. Concerning tumors with few mRNA we could do the same hypothesis if the weak positivity is supposed to be due to normal cell contamination. We could also hypothesize that the absence of MED12 protein in tumors with mRNA positivity could be due to post-transcriptional or translational inhibition by miRNA for example. According to the TargetScan microRNA target prediction algorithm [33] *MED12* 3'UTR presents potential target sites for miRNAs.

Interestingly concerning uterine LMS, *MED12* expression seems to be inhibited regardless the allele status. Indeed for the two mutated LMS, we could not know if the *MED12* mutation occurred on the inactivated X chromosome and if the second wild-type allele was transcriptionnally inactivated or if it's the activated X allele which was mutated and then subsequently inhibited. However, collectively these results show that *MED12* may be implicated in the early steps of both benign and malignant uterine tumor development, its expression being inhibited in a subset of tumors, those with malignant potential.

Array-CGH data show that the inhibition of *MED12* expression is associated with malignant tumors. Actually, benign tumors are generally associated with simple genomic profiles [23], [34]–[36], whereas most malignant tumors exhibit much altered profiles and these are tumors in which the number and type of genetic alterations are strong prognostic factors [37]–[38]. In uterine smooth muscle tumors it has been described that uterine LMS, as LMS from internal trunk and limbs, exhibit highly rearranged genomic profiles, while LM present no or few alterations detected by array-CGH [23]–[24]. In our series, all tumors that expressed *MED12* mutations exhibited no or very few genomic alterations. The only two tumors with a *MED12* mutation and a rearranged genomic profile were LMS, which also exhibited complete *MED12* expression inhibition. In the same manner, all tumors without *MED12* expression presented altered genomic profiles. As a result, even if *MED12* mutations are not restricted to tumors without genomic alterations, it seems that inhibition of its expression is specific to malignant rearranged uterine tumors. Thus, we could hypothesize that *MED12* mutations have been acquired before malignant transformation. Its expression loss could occur later in the malignant transformation process or could correspond to another mechanism of *MED12* inactivation specific of rearranged tumors.

Makinen *et al*. have suggested based on bioinformatics pathway analysis [17] that *MED12* mutations could be involved in LM development through activation of the Wnt/β-catenin pathway; the Wnt/β-catenin target genes being among the genes positively regulated by MED12 [25]–[26]. However a recent study combining mRNA and miRNA differential expression between LM and myometrium has observed a downregulation of the Wnt pathway and an upregulation of the focal adhesion pathway in LM [39]. Our β-catenin immunohistochemistry data tends to indicate that the canonical Wnt pathway is not implicated in LM development since β-catenin, when expressed, is located to the membrane in mutated cases (5/7 cases = 71.4%); a localization which has been demonstrated to be indicative of a low transactivation activity [40]–[41]. The Wnt/β-catenin pathway does not seem constitutively activated in these mutated tumors and we could thus hypothesize that if *MED12* mutations play a role in uterine tumor development it's probably not through Wnt target genes activation in association with β-catenin. In order to precisely assess pathways which could be activated by *MED12* mutations it seems necessary to compare expression profiles between mutated LM and non-mutated LM.

When we consider each histotype separately, we see that positive cytoplasmic β-catenin labeling, associated or not with membranous staining, is observed in 22.2% of classical LM, 20% of atypical LM, 37.5% of STUMP and 40% of LMS. This means that even if the β-catenin membranous labeling is predominant in all uterine tumors subtypes, the frequency of β-catenin cytoplasmic localization tends to increase in parallel with tumor malignity. These results suggest that the Wnt pathway could be implicated in malignant progression, probably without MED12 involvement. Data is scarce in the literature regarding the β-catenin localization in uterine tumors but two studies previously reported no nuclear staining [42]–[43], results consistent with ours. Conversely nuclear β-catenin labeling has been previously observed in 23% of uterine LMS, membranous staining in 25% of LMS and cytoplasmic positivity in 36% of LMS [44]. Discrepancies between the studies concerning LMS may be due to the size of the series (245 LMS versus 10), to the antibody used or to the labeling interpretation. β-catenin functional models may be useful to study its role in these tumors.

Another question of great interest is the similarity between uterine LMS and LMS from other locations. On the basis of our results we have observed that LMS from internal trunk and limbs do not exhibit *MED12* mutations and that they all express *MED12*. *MED12* alterations seem to be specific to a subgroup of uterine malignant tumors. *MED12* expression loss may contribute to the oncogenesis process of this subset of uterine tumors but not to LMS from other locations, meaning that these tumors could be two different entities or at least originating from distinct genetics and/or cell types. It could be interesting to analyze in further detail the two groups of uterine LMS, *i.e*. those with and those without *MED12* alterations, in order to see if they also represent distinct entities and if the LMS group without *MED12* alterations is closer to the non-uterine LMS group than to the other uterine group. In the same manner, it could be of great interest to study non-uterine LM to see if *MED12* alterations are really exclusive to uterine tumors.

The role of *MED12* in the oncogenesis process has not already been assessed and it may be hard to determine because of its both repressive and activating functions according to the cellular context as reviewed in [18]–[19]. In Makinen *et al*.'s study, *MED12* mutations observed in LM are supposed to be activating ones [17]. Substantial data supports this idea: the absence of nonsense mutations, the presence of in-frame deletions and the predominant mutated allele expression. However McGuire *et al*. proposed the opposite hypothesis [22]. According to the transcriptional repressive and chromatin modifying known functions of *MED12*, they hypothesize that *MED12* could be a tumor suppressor gene, leading to abnormal leiomyomatous growth when mutated. This hypothesis of tumor suppressor gene is strengthened by our data showing that a subgroup of rearranged tumors exhibits a loss of MED12 expression. We could thus hypothesize that *MED12* mutations modify or attenuate a function of the protein leading to a benign proliferation and that only the loss of all MED12 functions by expression inhibition could be implicated in malignant transformation. This hypothesis is supported by the fact that all mutations affect the same domain of the gene; all deletions are in-frame and are expressed at mRNA and protein levels suggesting that other domains of the protein could still be functional in LM.

We report here the first *MED12* mutations and expression alteration in uterine LMS. It is now essential to validate our hypotheses regarding the role of *MED12* mutations in leiomyoma development and MED12 inhibition in leiomyosarcomas oncogenesis. To address these issues we plan to modulate MED12 expression in uterine LM and LMS cell lines. Further investigations, establishing murine models with specific knock-out of MED12 in smooth-muscle cells as performed for example with connexin 43 [45] as well as analyzing the impact of a *MED12* mutated allele expression or a *MED12* knock-out allele in mesenchymal stem cells, fibroblasts or smooth-muscle cells may be also useful to validate the model.

## Materials and Methods

### Ethics Statement

The samples used in this study as part of the Biological Resources Center of Bergonie Cancer Institute (CRB-IB). Accordance with the French Public Health Code (articles L. 1243-4 and R. 1243-61), the CRB-IB has received the agreement from the French authorities to delivered samples for scientific research (number AC-2008-812, on February 2011). These samples are from care and requalified for research. The patients signed a consent approved by the Committee of Protection of Individuals.

### Samples and histology

A retrospective series of 33 cases of uterine SMT (9 LM, 5 atypical LM, 9 STUMP and 10 LMS) collected in the Department of Pathology of the Bergonie Cancer Institute in Bordeaux was reviewed by two pathologists with interest in gynecopathology (GMG and SC). According to Bell *et al.*'s criteria [4], a SMT without atypia, without necrosis and low mitotic count was diagnosed as LM (<5 mitosis/10 HPFs for epithelioid variant, <2 mitosis/10 HPFs for myxoid variant); atypical LM is a LM variant with atypical, unusual nuclei with spotty distribution and <10 mitosis/10 HPFs. A SMT with important and diffuse atypia and/or coagulative necrosis and high mitotic count was classified as LMS. We made the diagnosis of STUMP in the following histological patterns: 1) diffuse atypia (moderate to severe) with mitotic rate ≤10 mitosis/10 HPFs without necrosis; 2) focal atypia (moderate to severe) and >10 mitosis/10 HPFs without necrosis; 3) no to mild atypia with ≥20 mitosis/10 HPFs without necrosis; 4) no atypia ≤10 mitosis/10 HPFs with necrosis [46]. These tumors came from 32 individual patients. STUMP3 is the local recurrence of STUMP2. Clinical and pathological data are presented in Table S1.

The 38 cases of nongynecological LMS were reviewed by a pathologist expert in soft tissue sarcomas (JMC) according to the World Health Organization recommendations [47]. These tumors came from 38 individual patients. Clinical and pathological data are presented in Table S2.

### DNA and RNA extraction

For internal trunk LMS and LMS of the limbs, genomic DNA was isolated using a standard phenol-chloroform extraction protocol on frozen samples. For uterine SMT, which are paraffin-embedded tissues, genomic DNA was extracted according to Agilent protocol for DNA isolation on FFPE tissues (http://www.chem-agilent.com/pdf/G4410-90020v3_1_CGH_ULS_Protocol.pdf) (Agilent Technologies).

For RNA extraction, paraffin was removed using two steps in toluene followed by two steps in absolute ethanol. Samples were then incubated over-night at 55°C in 200 µl of ATL buffer (Qiagen DNeasy Blood & Tissue Kit, Qiagen) and 20 µl of proteinase K. Additional 10 µl of proteinase K were added twice the two next days and samples were incubated as previously described. Total RNA was then extracted using a standard TRIzol (Life Technologies)/chloroform extraction followed by an isopropanol precipitation. Finally, genomic DNA and RNA were quantified using a Nanodrop 1000 spectrophotometer (Thermo Scientific).

### Mutation Screening

Mutation screening of *MED12* exon2 was assessed on genomic DNA and on cDNA. For cDNA sequencing, total RNA was first reverse transcribed using High Capacity cDNA Reverse Transcription Kit (Applied biosystems) according to the manufacturer's instructions. Primers used were designed using Primer 3 program (http://frodo.wi.mit.edu/primer3/) and are presented in Table S3. Pre-sequencing PCR was realized on 50 ng of genomic DNA or cDNA using AmpliTaqGold® DNA polymerase (Applied Biosystems) with an annealing temperature of 60°C. PCR was also realized using *β2M* (β-2-migroglobulin) primers as control (Table S3). PCR products were then purified using ExoSAP-IT PCR Purification Kit (GE Healthcare) and sequencing reactions were performed with the Big Dye Terminator V1.1 Kit (Applied Biosystems) according to the manufacturer's recommendations. Samples were then purified using the Big Dye XTerminator Purification kit (Applied Biosystems) according to the manufacturer's instructions and sequencing was performed on a 3130xl Genetic Analyzer (Applied Biosystems). Sequences analysis was performed with SeqScape software v2.5 (Applied Biosystems). Mutations are referenced on the Wellcome Trust Sanger Institute webpage as part of the COSMIC project (Catalogue Of Somatic Mutations In Cancer) http://www.sanger.ac.uk/perl/genetics/CGP/cosmic?action=mutations&ln=MED12&sn=soft_tissue&hn=leiomyoma&start=1&end=2178&coords=AA:AA&neg=off&page=1.

### Array-CGH

Array-CGH experiments were thus realized for 30 cases with good enough DNA quality. No array-CGH results are available for LM11, STUMP2 and LMS3. DNA was first treated using a DNase as previously described [48]. DNA was then hybridized to 8×60K whole-genome Agilent arrays (G4450A) as previously described [38]. Microarray slides were scanned using an Agilent DNA Microarray Scanner, images were analyzed by Feature Extraction V10.1.1.1 and then analyzed by Agilent Genomic Workbench Lite Edition 6.5.0.18 (Agilent). The ADM-2 algorithm was used to identify DNA copy number anomalies at the probe level. A low-level copy number gain was defined as a log 2 ratio >0.25 and a copy number loss was defined as a log 2 ratio <−0.25. A high-level gain or amplification was defined as a log 2 ratio >1.5 and a homozygous deletion was suspected when the ratio was below −1.

### Immunohistochemistry

Tissue sections were obtained from paraffin blocks. Immunohistochemistry was performed using a Benchmark Ultra automated stainer (Ventana), a beta-catenin antibody (manufacturer's dilution, Clone 14, 760–4242, Ventana) and a MED12 antibody (dilution: 1/30, HPA003184-Ab1, Sigma) according to the manufacturer's recommendations. IHC pictures were taken using a Leitz DMRB microscope (Leica) and a DS-Ri1 camera (Nikon).

## Supporting Information

Table S1 **Uterine smooth muscle tumors clinical and pathologic data.** Histotype, localization and size of the 33 SMT are indicated in this table. Age of the patients at the diagnosis is also mentioned. Data availability for the different techniques used is indicated for each tumor. LM: leiomyoma; STUMP: Smooth muscle Tumor of Uncertain Malignant Potential; LMS: leiomyosarcoma; A: available; NA: not available.
(DOC)

Table S2 **Non-uterine leiomyosarcomas clinical and pathologic data. L**ocalization and size of the 38 non-uterine LMS are indicated in this table. Age and sex of the patients are also mentioned. Data availability for the different techniques used is indicated for each tumor. LMS: leiomyosarcoma; A: available; NA: not available.
(DOC)

Table S3 **Primers used.** MED12 and β2-microglobulin forward and reverse primers are presented.
(DOC)
