# Supplementary material for: MED12 Alterations in Both Human Benign and Malignant Uterine Soft Tissue Tumors
Source: PLoS One. 2012 Jun 29;7(6):e40015. doi: 10.1371/journal.pone.0040015 (PMC3386951; doi:10.1371/journal.pone.0040015)
Supplement: Table S1 — Uterine smooth muscle tumors clinical and pathologic data. Histotype, localization and size of the 33 SMT are indicated in this table. Age of the patients at the diagnosis is also mentioned. Data availability for the different techniques used is indicated for each tumor. LM: leiomyoma; STUMP: Smooth muscle Tumor of Uncertain Malignant Potential; LMS: leiomyosarcoma; A: available; NA: not available. (DOC) [file pone.0040015.s001.doc]

| **Tumor** | **Histotype** | **Age** | **Localization** | **Size** | **Array** | **Sequencing** | **IHC** |
| --- | --- | --- | --- | --- | --- | --- | --- |
| **Name** |  |  |  | **(mm)** | **CGH** | **gDNA/cDNA** |  |
| LM1 | LM | 51 | Uterus | 65 | A | A | A |
| LM2 | LM | 65 | Uterus | 14 | A | A | A |
| LM3 | LM | 46 | Retroperitoneum | 35 | A | A | A |
| LM4 | LM | 49 | Uterus | 150 | A | A | A |
| LM5 | LM | 52 | Uterus | 33 | A | A | A |
| LM6 | LM | 41 | Uterus | 40 | A | A | A |
| LM7 | LM | 50 | Uterus | 37 | A | A | A |
| LM8 | LM | 47 | Uterus | 40 | A | A | A |
| LM9 | LM | 68 | Broad ligament | 70 | A | A | A |
| LM10 | Atypical LM | 60 | Uterus | 420 | A | A | A |
| LM11 | Atypical LM | 47 | Uterus | 70 | NA | A | A |
| LM12 | Atypical LM | 51 | Uterus | NA | A | A | A |
| LM13 | Atypical LM | 52 | Uterus | NA | A | A | A |
| LM14 | Atypical LM | 49 | Uterus | 55 | A | A | A |
| STUMP1 | STUMP | 38 | pelvis | 95 | A | A | A |
| STUMP2 | STUMP | 84 | Left ovary | NA | NA | A | NA |
| STUMP3 | STUMP | 84 | Pelvis | 170 | A | A | A |
| STUMP4 | STUMP | 55 | Uterus | NA | A | A | A |
| STUMP5 | STUMP | 42 | Uterus | 70 | A | A | A |
| STUMP6 | STUMP | 37 | Uterus | 140 | A | A | A |
| STUMP7 | STUMP | 75 | Uterus | 120 | A | A | A |
| STUMP8 | STUMP | 32 | Pelvis | 170 | A | A | A |
| STUMP9 | STUMP | 50 | Uterus | 190 | A | A | A |
| LMS1 | LMS | 65 | Uterus | 100 | A | A | A |
| LMS2 | LMS | 59 | Uterus | 140 | A | A | A |
| LMS3 | LMS | 66 | Uterus | 30 | NA | A | A |
| LMS4 | LMS | 80 | Uterus | 120 | A | A | A |
| LMS5 | LMS | 62 | Uterus | 28 | A | A | A |
| LMS6 | LMS | 62 | Uterus | 120 | A | A | A |
| LMS7 | LMS | 45 | Uterus | 65 | A | A | A |
| LMS8 | LMS | 73 | Uterus | 20 | A | A | A |
| LMS9 | LMS | 73 | Uterus | 300 | A | A | A |
| LMS10 | LMS | 70 | Uterus | 130 | A | A | A |

**Table S1**
