# Supplementary material for: MED12 Alterations in Both Human Benign and Malignant Uterine Soft Tissue Tumors
Source: PLoS One. 2012 Jun 29;7(6):e40015. doi: 10.1371/journal.pone.0040015 (PMC3386951; doi:10.1371/journal.pone.0040015)
Supplement: Table S2 — Non-uterine leiomyosarcomas clinical and pathologic data. Localization and size of the 38 non-uterine LMS are indicated in this table. Age and sex of the patients are also mentioned. Data availability for the different techniques used is indicated for each tumor. LMS: leiomyosarcoma; A: available; NA: not available. (DOC) [file pone.0040015.s002.doc]

| **Tumor** | **Sex** | **Age** | **Localization** | **Size** | **Sequencing** | **RT-PCR** |
| --- | --- | --- | --- | --- | --- | --- |
| **Name** |  |  |  | **(mm)** | **gDNA** |  |
| LMS30 | F | 69 | Chest wall | 80 | A | A |
| LMS47 | M | 45 | Thorax internal | 150 | A | A |
| LMS31 | F | 73 | Groin | 70 | A | A |
| LMS32 | F | 68 | Retroperitoneum | 200 | A | A |
| LMS33 | F | 47 | Retroperitoneum | NA | A | A |
| LMS34 | M | 75 | Thigh | 130 | A | A |
| LMS35 | M | 28 | Buttock | 80 | A | A |
| LMS36 | F | 64 | Pelvis | NA | A | A |
| LMS37 | M | 57 | Thigh | 70 | A | A |
| LMS38 | M | 39 | Thigh | 100 | A | A |
| LMS48 | M | 88 | Thigh | 80 | A | A |
| LMS11 | F | 55 | Thigh | 50 | A | A |
| LMS12 | M | 70 | Chest wall | 100 | A | A |
| LMS13 | M | 35 | Leg | 100 | A | A |
| LMS14 | M | 58 | Knee | 100 | A | A |
| LMS15 | M | 73 | Thigh | 80 | A | A |
| LMS16 | M | 46 | Retroperitoneum | 120 | A | A |
| LMS17 | F | 75 | Thigh | 110 | A | A |
| LMS18 | F | 46 | Abdominal wall | 120 | A | A |
| LMS19 | F | 92 | Upper arm | 80 | A | A |
| LMS20 | F | 77 | Thigh | 110 | A | A |
| LMS21 | M | 54 | Thigh | 100 | A | A |
| LMS22 | M | 62 | Retroperitoneum | 100 | A | A |
| LMS23 | M | 69 | Thigh | 130 | A | A |
| LMS24 | F | 42 | Retroperitoneum | 150 | A | A |
| LMS25 | M | 71 | Thigh | 50 | A | A |
| LMS26 | F | 37 | Thigh | 40 | A | A |
| LMS27 | F | 83 | Upper arm | 100 | A | A |
| LMS28 | M | 54 | Buttock | 120 | A | A |
| LMS29 | F | 60 | Retroperitoneum | 60 | A | A |
| LMS39 | F | 66 | Thigh | 150 | A | A |
| LMS40 | M | 66 | Retroperitoneum | 110 | A | A |
| LMS41 | F | 69 | Thigh | 80 | A | A |
| LMS42 | M | 78 | Elbow | 80 | A | A |
| LMS43 | F | 72 | Chest wall | 160 | A | A |
| LMS44 | F | 67 | Retroperitoneum | 100 | A | A |
| LMS45 | F | 59 | Retroperitoneum | 32 | A | NA |
| LMS46 | F | NA | Thigh | 85 | A | NA |

**Table S2**
