# Supplementary material for: MED12 Alterations in Both Human Benign and Malignant Uterine Soft Tissue Tumors
Source: PLoS One. 2012 Jun 29;7(6):e40015. doi: 10.1371/journal.pone.0040015 (PMC3386951; doi:10.1371/journal.pone.0040015)
Supplement: Table S3 — Primers used. MED12 and β2-microglobulin forward and reverse primers are presented. (DOC) [file pone.0040015.s003.doc]

|  | **Forward primer** | **Reverse primer** |
| --- | --- | --- |
| ***MED12* gDNA** | 5’-CCCTACTCTCCCACCCCTTC-3’ | 5’-CTTCAGCCTGGCAGAGTTGT-3’ |
| ***MED12* cDNA** | 5’-GGCCTCCCGATGTTTACC-3’ | 5’-AAGCTGACGTTCTTGGCACT-3’ |
| ***β2M***  **cDNA** | 5’-TGACTTTGTCACAGCCCAAGATA-3’ | 5’-AATCCAAATGCGGCATCTTC-3’ |

**Table S3**
